# Cysteine-Rich LIM-Only Protein 4 (CRP4) Promotes Atherogenesis in the ApoE^−/−^ Mouse Model

**DOI:** 10.3390/cells11081364

**Published:** 2022-04-17

**Authors:** Natalie Längst, Julia Adler, Anna Kuret, Andreas Peter, Peter Ruth, Karsten Boldt, Robert Lukowski

**Affiliations:** 1 Department of Pharmacology, Toxicology and Clinical Pharmacy, Institute of Pharmacy, University of Tübingen, 72076 Tübingen, Germany; natalie.zinn@uni-tuebingen.de (N.L.); julia.straubinger@uni-tuebingen.de (J.A.); anna.kuret@uni-tuebingen.de (A.K.); peter.ruth@uni-tuebingen.de (P.R.); 2 Department for Diagnostic Laboratory Medicine, Institute for Clinical Chemistry and Pathobiochemistry, University Hospital Tübingen, 72076 Tübingen, Germany; andreas.peter@med.uni-tuebingen.de; 3 Molecular Biology of Retinal Degenerations, Institute of Ophthalmic Research, University of Tübingen, 72076 Tübingen, Germany; karsten.boldt@uni-tuebingen.de

**Keywords:** acyl-CoA dehydrogenase long chain (ACADL), apolipoprotein E (ApoE), 3′,5′-cyclic guanosine monophosphate (cGMP), 3′,5′-cyclic guanosine monophosphate-dependent protein kinase type I (cGKI), cysteine-rich LIM-only protein 4 (CRP4), microtubule associated monooxygenase, Calponin and LIM domain containing 2 (MICAL2), pirin (PIR), peroxiredoxin-4 (PRDX4)

## Abstract

Vascular smooth muscle cells (VSMCs) can switch from their contractile state to a synthetic phenotype resulting in high migratory and proliferative capacity and driving atherosclerotic lesion formation. The cysteine-rich LIM-only protein 4 (CRP4) reportedly modulates VSM-like transcriptional signatures, which are perturbed in VSMCs undergoing phenotypic switching. Thus, we hypothesized that CRP4 contributes to adverse VSMC behaviours and thereby to atherogenesis in vivo. The atherogenic properties of CRP4 were investigated in plaque-prone apolipoprotein E (ApoE) and CRP4 double-knockout (dKO) as well as ApoE-deficient CRP4 wildtype mice. dKO mice exhibited lower plaque numbers and lesion areas as well as a reduced content of α-smooth muscle actin positive cells in the lesion area, while lesion-associated cell proliferation was elevated in vessels lacking CRP4. Reduced plaque volumes in dKO correlated with significantly less intra-plaque oxidized low-density lipoprotein (oxLDL), presumably due to upregulation of the antioxidant factor peroxiredoxin-4 (PRDX4). This study identifies CRP4 as a novel pro-atherogenic factor that facilitates plaque oxLDL deposition and identifies the invasion of atherosclerotic lesions by VSMCs as important determinants of plaque vulnerability. Thus, targeting of VSMC CRP4 should be considered in plaque-stabilizing pharmacological strategies.

## 1. Introduction

Atherosclerosis is a multifactorial vascular disease affecting large arteries [1,2]. It can be regarded as a chronic inflammatory process involving multiple cell types, including monocytes, macrophages, lymphocytes and vascular smooth muscle cells (VSMCs), which collectively contribute to the initiation and formation of atherosclerotic plaques [3]. VSMCs represent the major cell type of the tunica media in arteries and veins [4], and in their contractile state, they contribute to vascular tone and vessel diameter [4,5]. Dedifferentiation into a synthetic phenotype, which is called phenotypic modulation or “switching”, is a crucial mechanism by which VSMCs respond to vessel wall injury and to the pathogenesis of atherosclerosis [6]. The rupture of an atherosclerotic plaque leads to luminal thrombosis and, depending on the thrombus burden and the degree of vessel occlusion, to myocardial infarction (MI) and stroke as its major clinical consequences [7,8]. The crucial risk factors for atherosclerosis are hyperlipidaemia with obesity, hypertension, and type 2 diabetes [9,10]. The onset of atherogenesis is further marked by the accumulation and oxidation of low-density lipoprotein (LDL) in the subendothelial space, involving endothelial cell damage and dysfunction [11,12]. This leads to the attraction and diapedesis of monocytes followed by their transformation to macrophages, which take up oxidized LDL (oxLDL) particles and thereby become foam cells. VSMCs invade the vessel intima where their proliferative state promotes plaque progression [1,2,4,8,13], while VSMC-derived cells in advanced lesions may also contribute to the formation of a stable fibrous cap and thus to stability [2]. Once formed, multiple pathophysiological mechanisms provoke the destabilization of advanced plaques. Key among these are the hypersecretion of matrix-degrading proteases [14], oxidative stress [15] and the necrotic and apoptotic cell death of macrophages and VSMCs [16,17]. The degree of VSMC plasticity in atherosclerosis has been largely underestimated in the past. A significant number of cells in atherosclerotic plaques of VSMC origin do not express classical contractile markers, such as α-smooth muscle actin (α-SMA) [18], while acquiring properties of macrophages and foam cells [13], mesenchymal stem cells, osteochondrogenic cells or extracellular-matrix-producing cells [3,8]. This implies that at least some subsets of VSMC-derived cells display a high potential for transdifferentiation [13,19]. Hence, VSMC survival, the proliferative, migratory, and secretory capacity of these cells as well as their ability to transdifferentiate into multiple plaque cell types play major, distinct and/or sometimes opposing roles for the formation of vascular lesions. Given this extensive evidence, a selective manipulation of the pro-atherogenic VSMC factors and/or behaviours should possess great potential for plaque-stabilizing interventions [20].

Previously, it was supposed that the cysteine-rich LIM-only protein 4 (CRP4 *aka* CRP2) exhibits regulatory functions on cell plasticity in VSMC-like cells [21]. CRP4 contains two LIM domains, consisting of tandemly arranged zinc finger structures [22] where the zinc ions are complexed by highly conserved cysteine- or histidine-rich residues. The amino acid (aa) sequence connecting the two LIM domain containing zinc fingers has been considered as a flexible hinge or linker region of the protein [23], while the flanking LIM domains act as scaffolding modules. These modules mediate protein–protein interactions between various classes of signalling molecules, transcription factors and structural proteins allowing locally controlled assembly of multimeric protein complexes [24,25].

Thus far, three related proteins of CRP4 (*Crip2*), which is also known as heart LIM protein (HLP) [21,26,27,28,29], have been identified within the CRP family: CRP1 (*Csrp1*) [30], CRP2/smLIM (*Csrp2*) [31] and CRP3/MLP (*Csrp3*) [32]. The gene symbol for CRP4 differs from the standard nomenclature of the CRP genes because it is closely related to but also distinct from the other members of this family. This is also reflected in the extent of the aa sequence identity between CRP1-3 versus CRP4, which varies from 35% up to 80% [27]. Despite such substantial differences in homology, all members seem to determine cell lineage, and they have been implicated as critical regulators of differentiation in various cell types including VSMCs [33,34,35]. For example, a lack of CRP2/smLIM enhanced VSMC migration and increased neointima formation following wire-induced arterial injury [34], while the opposite was seen for CRP1/*Csrp1*-deficient mice [33]. Interestingly, the arterial injury phenotype produced by CRP1/CRP2 double-knockout mice was identical to the response of respective wildtype (WT) animals suggesting that CRP1 and CRP2, in a functionally antagonistic manner, regulate VSMC activity in response to vascular injury [33].

Of note, CRP4 is the only LIM-domain-containing CRP family member that contains a unique RKTS^104^ motif in its linker region. With the Ser^104^ residue, this consensus sequence possesses a site for phosphorylation by 3′,5′-cyclic guanosine monophosphate (cGMP)-dependent protein kinase type I (cGKI), a kinase that mediates vascular nitric oxide (NO)- and natriuretic peptide (NP)-induced cGMP signals [23]. In addition to regulating vascular tone and endothelial permeability [36,37], the cGMP pathway has been implicated to play crucial roles for VSMC proliferation in vitro and for atherogenesis in vivo [38,39,40,41]. Whether any of these in vivo effects attributed to the NO- or NP-stimulated cGMP axis in VSMCs involve CRP4 remains largely unclear at present. It is known, however, that CRP4 cooperatively interacts with the transcription factors serum response factor (SRF) and GATA6 in the α-SMA promoter region of VSMC-like cells. In this ex-vivo setting, the phosphorylation of CRP4 at Ser^104^ (pSer^104^) was implicated in the cGMP/cGKI-mediated effects on SM-specific gene transcript expression [21,23,42]. The putative roles of CRP4 in vasculo-proliferation and atherosclerosis, however, warrant further investigation.

In this study, we investigated the atherogenic properties of CRP4 using plaque-prone ApoE and CRP4 double-knockout (dKO) mice (ApoE^−/−^/CRP4^−/−^). Double KO mice and their respective ApoE-deficient CRP4 WT littermates (ApoE^−/−^/CRP4^+/+^) were subjected to an atherogenic diet to analyse the development and composition of the plaques in vivo. These approaches were corroborated by assessing the migratory and proliferative properties of primary and highly passaged CRP4 WT and KO VSMCs and by correlating the putative changes in cell plasticity with the VSMC proteome.

## 2. Material & Methods

### 2.1. Animal Care

All experiments with animals were authorized by the local ethics Committee for Animal Research (Regierungspräsidium Tübingen, Germany) and were performed in accordance with the German Animal Welfare Act. The animals were kept in standardized cages on a 12 h light/dark-cycle, and the housing conditions were temperature- and humidity-controlled. We ensured that the animals had *ad libitum* access to food (Altromin, Lage, Germany; Sniff Spezialdiäten GmbH, Soest, Germany) and water. For the ex vivo experiments, VSMCs were obtained from global CRP4 knockout (CRP4 KO; genotype: CRP4^−/−^) and their wildtype littermates (CRP4 WT; genotype: CRP4^+/+^) maintained on an inbred 129Sv background [27,42]. For the in vivo atherosclerosis study, heterozygous CRP4 mice (genotype: ApoE^−/−^/CRP4^+/−^) lacking ApoE (C57BL6N) were generated and mated to each other to establish CRP4 and ApoE dKO animals (genotypes: ApoE^−/−^/CRP4^−/−^ *aka* dKO) as well as CRP4 WT and ApoE KO (genotypes: ApoE^−/−^/CRP4^+/+^) from the same litters on a mixed C57BL6N/129Sv background. Western diet (WD, 0.21% Cholesterol, which corresponds to TD.88137; Sniff Spezialdiäten GmbH, Soest, Germany) was fed as an atherogenic diet for either 8 or 16 weeks, while groups of control mice received a control diet (CD, which corresponds to CD.88137; Sniff Spezialdiäten GmbH, Soest, Germany). All experimental mice were bred and maintained at the Institute of Pharmacy (Department of Pharmacology, Toxicology and Clinical Pharmacy; Experimental Pharmacology) at the University of Tübingen as described before [27,29]. In vivo feeding studies were conducted with two independent cohorts of male mice, while data from both sexes were pooled for in vitro cell growth and migration experiments.

### 2.2. In Vivo Atherosclerosis Mouse Model, Aorta Isolation and Plasma Lipid Profiles

At the age of 5 weeks, ApoE^−/−^/CRP4^+/+^ and dKO mice were fed for 8 or 16 weeks with WD or CD. The body weight (BW) of the mice was documented twice a week and for the glucose, triglyceride and cholesterol measurements, blood samples were taken every fourth week from the tail vein. After 8 or 16 weeks of the diets, the final BW was determined prior to euthanasia with CO_2_.

Subsequent steps followed the protocol by Mohanta et al. [43]. In brief, the death of the mice was confirmed, and the abdomen of the mouse was opened to collect a final blood sample from the right atrium using a 1 mL syringe with 23 G cannula. To separate the plasma, whole blood samples were centrifuged at 2000× *g* for 5 min at 4 °C, and the supernatant was frozen away at −20 °C. The analysis of cholesterol, LDL, HDL, triglyceride and glucose was conducted using the automated clinical chemistry analyser ADVIA XPT Clinical Chemistry System (Siemens Healthineers, Eschborn, Germany). The amount of VLDL was calculated by subtracting the LDL and HDL from the whole cholesterol level. Next, in situ perfusion via a left ventricular puncture was performed using 10 mL ice-cold EDTA (5 mM in phosphate-buffered saline (PBS)) following 20 mL ice-cold PBS allowing tissue dissection for further processing. Subsequently, the organs were removed, and the aorta was thoroughly cleaned from fat and adventitial tissue from the heart to the iliacal bifurcation.

### 2.3. En Face Oil Red O Staining

For Oil Red O staining, perfusion through the left ventricle was performed with 5 mL 4% PFA in PBS followed by 10 mL 5% sucrose in PBS 10 min later. Thereafter, the aorta from the heart to the iliacal bifurcation was dissected and placed in a petri dish with a black wax cover in PBS. The remaining fatty tissue around the aortic vessels was carefully removed before the aorta was cut and pinned to the waxed underground in PFA-sucrose (5 g sucrose in 4% PFA in PBS, pH 7.4) overnight at room temperature. The next day, the aorta was washed three times with PBS and then incubated for 5 min with 60% isopropanol. Upon isopropanol removal, the aorta was incubated for 30 min with the Oil Red O solution (0.5 g Oil Red O dissolved in 100 mL isopropanol and diluted with 67 mL ddH_2_O) and afterwards washed three times for 2 s with 60% isopropanol and finally covered with ddH_2_O. For analysis of the plaque areas and total plaque burden Oil Red O stains were documented using a microscope camera from Distelkamp-Electronic and evaluated using the AxioVision Rel.4.8 software tool (Carl Zeiss AG, Oberkochen, Germany).

### 2.4. Cryosectioning of Aortic Vessels

Cryosectioning was performed on the aorta from their basis (three valve area) through all main branches of the aortic arch. This segment was incubated in 4% PFA in PBS for 4 h at 4 °C on a shaker and afterwards washed three times with PBS. To withdraw the remaining water, a sucrose gradient ranging from 5% to 20% sucrose was applied to the tissue for 36 h in total. Next, the aortic segments were embedded in NEG-50™ (Thermo Fisher Scientific, Waltham, MA, USA) and frozen at −80 °C. With a cryotome (Thermo Fisher Scientific) 8 µm thick cryosections were cut at −20 °C, collected on microscope slides (Thermo Fisher Scientific) and stored at −20 °C.

### 2.5. Immunohistochemistry and Immunofluorescence

Alkaline phosphatase (Vectastain ABC-AP-Kit Standard Alkaline Phosphatase, Vector laboratories, Burlingame, CA, USA) and immunofluorescence (IF) staining was conducted to identify multiple target proteins in the aorta, plaques and isolated VSMCs. For this purpose, aortic cryosections or VSMCs fixed for 10 min in 4% PFA were permeabilized with 0.3% Triton-X 100 in PBS for 15 min. Tissue and cells were then rinsed with PBS, and unspecific antibody-binding sites were blocked by applying 10% NDS in PBS for 1 h at room temperature according to previously published protocols [39,44]. After, the following primary antibodies were used for immune detection overnight at 4 °C: CRP4 (1:2000 dilution [42]), α-SMA (1:1000 dilution, abcam, Cambridge, UK), Ki-67 (1:1000 dilution, Cell Signaling Technology, Danvers, MA, USA), Ggps1 (1:500 dilution, Proteintech Europe, Manchester, UK), Rai14 (1:500 dilution, Proteintech Europe), Acadl (1:500 dilution, Proteintech Europe), PRDX4 (1:500 dilution, Proteintech Europe), Cu^2+^-oxLDL (1:500 dilution, Merck Millipore, Burlington, MA, USA) and the fluorophore coupled antibody α-SMA 647™ (1:50 dilution, Cell Signaling Technology). AlexaFluor^®^488 goat anti-rabbit antibodies (1:800 dilution, Life Technologies, Thermo Fisher Scientific) were used to visualize primary-antibody-antigen complexes by IF, while biotinylated goat anti-rabbit antibodies (1:500 dilution, Vector laboratories) were used for immunohistochemistry and cytochemistry. After 1 h in the dark at room temperature, sections or cells prepared for fluorescence microscopy were cover-slipped with PermaFluor (Thermo Fisher Scientific) containing the cell nucleus marker Hoechst (1:1000 dilution). The detection of the fluorescence staining was performed with a Carl Zeiss Axio Imager m2 connected with an Apotome 2.0 Slider. Alkaline phosphatase staining sections and cells were incubated for 1 h in biotinylated antibodies and thereafter for 30 min in the dark in ABC-AP-Reagent (5 mL PBS with Reagent A&B, Vector Laboratories) according to the manufacturer’s recommendations. AP staining was developed with AP-Substrate provided by the Vectastain ABC-AP-Kit (5 mL 100 mM Tris-HCl pH 8.2 with Reagent 1, 2 and 3 as well as Levamisol, Vector Laboratories). Phosphatase activity was stopped by rinsing sections and cells with tap water before samples were embedded with the aqueous mounting media Aquatex^®^ (VWR, Bruchsal, Germany) and scanned with the Pannoramic Flesh DESK DX (Sysmex Deutschland GmbH, Norderstedt, Germany).

### 2.6. Histological Staining of Aortic Lesions

Haematoxylin and eosin (H&E) staining was performed on aortic cryosections mounted on glass slides as previously described [39]. Upon a stepwise rehydration in decreasing concentrations of ethanol (100 to 50% in steps of 10–20%), the sections were washed three times for 2 min with ddH_2_O and then incubated in Harris Haematoxylin solution for 5 s (Carl Roth GmbH & Co. KG, Karlsruhe, Germany). Excessive staining solution was removed by rinsing the sections in tap water before 0.1% ammonia solution was used to intensify the blue colour in the nuclei. After rinsing the sections again in tap water, the samples were incubated for 10 min with the acidic Eosin-Y solution (Sigma Aldrich, München, Germany). The staining solution was removed with tap water before the samples were dehydrated in 80% and 100% ethanol. Dehydration was followed by a final clearing step using toluol (Carl Roth GmbH & Co. KG, Karlsruhe, Germany) for 5 min. After air-drying at room temperature, sections were embedded with the non-aqueous mounting medium DPX (Merck Millipore). Calcium deposits in atherosclerotic plaques were visualized by the Alizarin staining method [45]. Aortic cryosections were incubated in 100%, 90% and 70% ethanol and then briefly washed in ddH_2_O water before adding 2% Alizarin Red solution (Sigma Aldrich, München, Germany) for 2 min. After two brief dehydration steps in acetone and acetone-xylene (1:1), the stained sections were cleared in xylol for 5 min, and upon air-drying at room temperature, the slides were mounted with the non-aqueous mounting medium DPX (Merck Millipore).

### 2.7. Isolation of VSMCs from Mouse Aorta

The in vitro analyses of VSMCs were performed with isolated VSMCs obtained from the aorta of global CRP4^−/−^ and CRP4^+/+^ mice from both sexes at the age of 6–8 weeks. After euthanasia by CO_2_ inhalation, the aorta was isolated and cleaned from adventitial tissue. VSMC cultures were established from four aorta per genotype, which were pooled for the enzymatic dissociation as described previously [44]. In brief, aorta were cut into ~1 mm segments and digested by Papain (0.7 mg/mL from Sigma Aldrich) for 1 h at 37 °C following by Hyaluronidase Type I (1 mg/mL) with Collagenase Type II (1 mg/mL) (both from Sigma Aldrich) for 25 min at 37 °C. Digestion was stopped with VSMC culture media (10% FBS, 5% Penicillin in DMEM Glutamax^®^ from Thermo Fisher Scientific), and the cells were centrifuged for 7 min at 300× *g*. Sedimented cells were resuspended in 1 mL VSMC culture media, counted using a Neubauer haemocytometer (Merck Millipore) and seeded in 6-well plates (Corning GmbH, Kaiserslautern, Germany) at a density of 5× 10^4^ cells/mL. The medium was changed every third day until the experiments with primary VSMCs or highly passaged cells (P10-15) were performed. For analyses of primary and passaged VSMC, results were obtained from multiple (at least three) independent isolates of each genotype.

### 2.8. mRNA Transcript Analysis

The total RNA was purified from cultivated VSMCs using the NucleoSpin RNA kit (Macherey-Nagel, Dueren, Germany) as described by the manufacturer. The RNA concentration and purity were determined by UV photometry with a NanoPhotometer (Implen, München, Germany) and 500 ng RNA in diethyl pyro carbonated (DEPC)-treated double distilled H_2_O was employed as a template to synthesize cDNA with the iScript cDNA Synthesis Kit (Bio-Rad Laboratories GmbH, München Germany). Upon conversion to cDNA, samples were diluted (1:10 in DEPC H_2_O) and amplified using the iQ SYBR Green Supermix (Bio-Rad Laboratories GmbH, Feldkirchen, Germany)) and the CFX Connect real-time PCR system (Bio-Rad Laboratories GmbH) with the following specific primers: CRP4 forward: 5′-ACG ATG GCC AGC CCT ACT-3′ and CRP4 reverse: 5′-TAG CTG CCC ACA GCA CCA-3′. As an endogenous control for the quantitative PCR reaction and for the relative quantification of CRP4 RT-PCR, hypoxanthine phosphoribosyl transferase (HPRT) was used as a housekeeping gene: HPRT forward: 5′-CAT TAT GCC GAG GATTTG GA-3′ and HPRT reverse: 5′-CCT TCA TGA CAT CTC GAG CA-3′.

### 2.9. Immunoblot Analyses

Western blot analyses were performed either with aorta or isolated VSMC protein lysates deriving from CRP4^−/−^ and CRP4^+/+^ mice or aorta from ApoE^−/−^/CRP4^+/+^ and dKO that received WD or CD for 16 weeks. Tissue or cells were homogenized and lysed with ice-cold SDS lysis buffer (20 mM Tris-HCl (pH 8.3), 0.67% SDS, 238 mM β-Mercaptoethanol, PMSF), denatured at 95 °C for 10 min and centrifuged for 10 min with 10,000 rpm. The protein concentration was measured via the Bradford method and adjusted to a concentration of 4 µg/µL. Laemmli buffer was added before the samples were again denatured for 10 min at 95 °C. Protein lysates (60 µg/lane) were separated depending on their molecular weight by 10–17.5% SDS-PAGE. Upon transfer to a PVDF membrane (Immobilon, Merck Millipore), unspecific binding sites of the membrane were blocked with 5% non-fat dry milk in Tris-buffered saline (20 mM) containing 0.05% Tween 20. Binding of the following primary antibodies was permitted overnight at 4 °C: CRP4 (1:2000 dilution [42]), αSMA (1:1000 dilution, abcam), Ggps1 (1:1000 dilution, Proteintech Europe), Rai14 (1:1000 dilution, Proteintech Europe), α-Adducin (1:500 dilution, Santa Cruz Biotechnology, Dallas, Texas, USA), γ-Adducin (1:1000 dilution, Santa Cruz Biotechnology), Pirin (1:500 dilution, Santa Cruz Biotechnology), PRDX4 (1:1000 dilution, Santa Cruz Biotechnology), Acadl (1:1000 dilution, Proteintech Europe), GAPDH (1:1000 dilution, Cell Signaling Technology), β-Actin (1:500 dilution, Santa Cruz Biotechnology) and HSP60 (1:1000 dilution, Santa Cruz Biotechnology).

Further, the binding of the fluorophore-coupled secondary antibodies (Cy5 anti-rabbit in a 1:2500 dilution or Cy3 anti-mouse in a 1:1500 dilution, ECL Plex, GE Life Sciences, Little Chalfont, UK) was performed for 1 h at room temperature. For the detection of the specific antigen–antibody complexes, a GE Healthcare Amersham Imager was used.

### 2.10. Migration Assay of Primary VSMCs

A wound healing assay was conducted using migration dishes containing a three-well silicone insert (Ibidi GmbH, Gräfelfing, Germany). Upon isolation, 1.5 × 10^5^ VSMCs from both genotypes were seeded and cultured until they grew confluent. For generating a migration gap, the three-well insert was removed, and cells in the different reservoirs were monitored every 24 h for 72 h using phase-contrast microscopy attached to a digital Axiocam MRc Rev.3 camera. To quantify the gap closure, pictures were repeatedly taken from exactly the same gap positions.

### 2.11. xCELLigence Proliferation Assay

The proliferation of VSMCs was measured using the xCELLigence method (E-plate^®^ 16 and xCELLigence RTCA DP, Real Time Cell Analyzer, OMNI Life Sciences, Bremen, Germany), which is an impedance-based method that allows the measurement of cell growth in real-time. Prior to the measurement, the cell numbers of freshly isolated as well as highly passaged synthetic VSMCs from CRP4 WT and KO aorta were rigorously determined. The cell seeding concentrations were 2 × 10^4^ cells/well for freshly isolated VSMCs and 2.5 × 10^3^ cells/well for highly passaged VSMCs in 100 µL VSMC culture media on E-plates. The background was determined for 15 min with 100 µL VSMC culture media without cells. Cell growth was monitored in real-time until the cell index reached a plateau (approximately 72 or 200 h for highly passaged and freshly isolated VSMCs, respectively). Experimental data was collected and processed using xCELLigence RTCA-Pro Software (Agilent, Santa Clara, CA, USA).

### 2.12. Proteome Analysis

Proteomic analyses were conducted from cultured CRP4 WT and CRP4 KO VSMCs. Before lysis, using 400 µL lysis buffer (0.5% Nonidet P-40, protease inhibitor cocktail (Complete Mini; Roche, Mannheim, Germany) and phosphatase inhibitor cocktails II and III (Sigma-Aldrich, München, Germany) in 30 mM Tris-HCl (pH 7.4), 150 mM NaCl and 5 mM EGTA), cells were serum deprived for 48 h to obtain uniform and reproducible conditions. Protein lysates were immediately snap-frozen and stored at −80 °C until further processing. Liquid chromatography–tandem mass spectrometry (LC-MS/MS) analysis was performed on trypsin-digested lysates as described previously [27]. Significantly regulated proteins (*t*-test *p* < 0.05, Significance B < 0.05) were subjected to gene enrichment analysis using GetGo ([46] and http://getgo.russelllab.org/, last accessed 23 March 2022).

### 2.13. Statistical Analyses

All data are expressed as the means ±SEM. To confirm a Gaussian distribution, the Shapiro–Wilk test and Kolmogorov–Smirnov tests were performed prior to the subsequent analyses. An unpaired Student’s *t*-test as well as two-way ANOVA were applied to identify between normally distributed data representing different groups. *p*-values of <0.05 were considered significant. All statistical analyses were performed using GraphPad Prism 9 (GraphPad Software, San Diego, CA, USA).

The proteomic data are expressed as the ratio of median values of five independent measurements of *n* = 5 animals per genotype. A paired *t*-test with permutation-based FDR determination as well as the significance B test with Benjamini–Hochberg-based FDR determination were calculated using the Perseus software [47].

Investigators measuring atherosclerotic lesion areas and cross-sectional area were blinded to the identity of the specimens.

## 3. Results

### 3.1. CRP4 Is Expressed in Medial VSMCs and in Atherosclerotic Lesions

**Figure 1:**
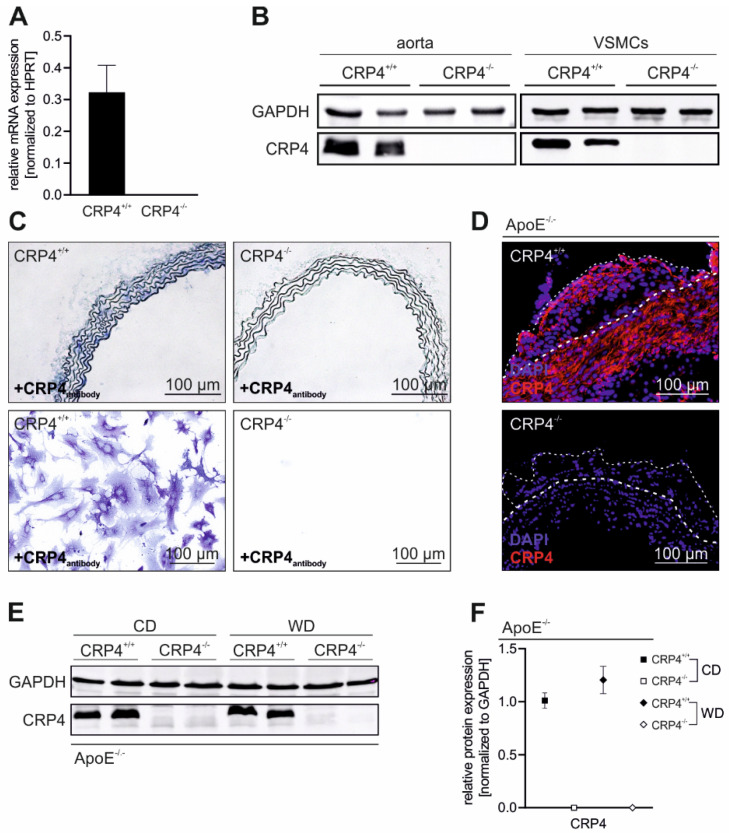
*CRP4 is expressed in VSMCs, in the tunica media and in atherosclerotic lesions*; (**A**) Quantitative polymerase chain reaction (qPCR) of CRP4 transcripts purified from isolated P0 VSMCs. (**B**) Western blot of the CRP4 protein expression in CRP4 WT and CRP4 KO aorta and in P10-15 VSMCs; *n* = 8 per genotype; data represent the mean ± SEM. Hypoxanthine phosphoribosyl transferase 2 (HPRT2) was co-amplified as a housekeeping gene in the qPCR approach (**A**) while glyceraldehyde-3-phospate dehydrogenase (GAPDH) was co-detected as a loading control in the Western blot analyses (**B**). (**C**) Representative CRP4 expression (blue) in CRP4 WT and KO aorta cryosections and in isolated P0 VSMCs by alkaline phosphatase (AP) staining. CRP4 (red) and DNA (Hoechst) were visualized (**D**) by immunofluorescence in aortic arch cryosections of 16-week WD-fed ApoE^−/−^/CRP4^+/+^ and dKO mice. Plaque and media margins are highlighted by thin and thick dashed lines (white), respectively. (**E**,**F**) Western blot and its quantification of CRP4 in ApoE^−/−^/CRP4^+/+^ versus dKO aortic lysates from WD and CD conditions. The CPR4 protein levels in WT samples were not altered by the diets, while the absence of CRP4 was again confirmed under WD and CD in dKO; *n* = 3–4 per genotype (every *n* was a pool of two aorta) and two-tailed Student’s *t*-test. All data are expressed as the means ± SEM.

To validate the vascular expression of CRP4 [21,23], we analysed the aorta and isolated VSMCs from CRP4-deficient and CRP4 WT mice [27,29]. At the transcript level in VSMCs (Figure 1A) and at the protein level in the aorta and VSMCs (Figure 1B), consistently high levels of CRP4 were detected in WT samples, whereas the aorta and VSMCs derived from KO animals remained CRP4 negative. CRP4 expression was present in the tunica media of the aorta and at a cellular level in primary CRP4 WT VSMCs. Immunohistochemical and cytochemical staining of CRP4 KO vessels and cells did not reveal CRP4-specific signals (Figure 1C). Next, we addressed whether CRP4 was present under pathophysiological conditions, i.e., in vascular lesions, frequently occurring in atherosclerosis-prone ApoE^−/−^ mice. Plaque formation was induced in ApoE^−/−^/CRP4^+/+^ and dKO mice receiving WD for 16 weeks (Supplementary Figure S1). In CRP4-proficient animals, CRP4 showed a broad expression pattern ranging from the tunica media beneath the aortic plaques to the inner lesion area and fibrous cap cells (Figure 1D), while again CRP4 was absent from dKO plaques.

These findings let us suppose a potential role of CRP4 for the formation/stabilization of atherosclerotic plaques in the ApoE^−/−^ mouse model. Importantly, the CRP4 expression did not differ between non-atherosclerotic (CD-fed mice) versus atherosclerotic (WD-fed mice) vessels (Figure 1E,F). This finding excluded aberrant effects of the diets on the vascular CRP4 abundance.

### 3.2. Lack of CRP4 Attenuates Atherosclerotic Plaque Progression in WD-Fed ApoE^−/−^ Mice

**Figure 2:**
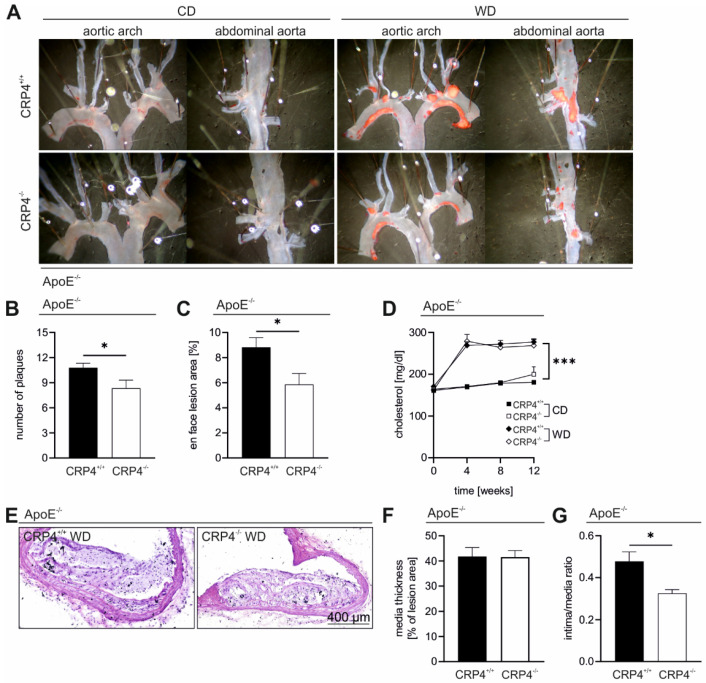
*Absence of CRP4 attenuated atherosclerotic plaque progression in the aorta of WD-fed* ApoE^−/−^. (**A**) En face Oil Red O staining of the aortic arch and the abdominal aorta of 16-week CD- vs. WD-fed ApoE^−/−^/CRP4^+/+^ and dKO mice. (**B**,**C**) Quantification of the plaque burden and size of the Oil Red O-stained areas within the plaques revealed a significantly decreased number of plaques and Oil Red O-stained atherosclerotic lesion area in ApoE^−/−^/CRP4^−/−^ mice compared to their littermate controls; *n* = 12–14 per genotype, *p < 0.05 and two-tailed Student’s *t*-test. (**D**) Cholesterol blood level of ApoE^−/−^ mice after 0, 4, 8 and 12 weeks were significantly elevated under WD versus CD conditions; *n* = 4–10 per group, *** *p* < 0.001 and two-tailed Student’s *t*-test. (**E**,**F**) Representative H&E-stained cross-sectional images prepared from atherosclerotic lesion area did not reveal differences in media thickness in the plaque region. (**G**) Consistent with the Oil Red O staining, the intima/media ratio in H&E-stained aorta cryosections of dKO aorta was significantly reduced; *n* = 6–7 per genotype, * *p* < 0.05 and two-tailed Student’s *t*-test. All data are expressed as the mean ± SEM.

To further examine the role of VSMC CRP4 in the pathogenesis of atherosclerosis, we examined the overall plaque burden and plaque size in ApoE^−/−^/CRP4^+/+^ and dKO mice. *En face* Oil Red O staining of dKO aorta (Figure 2A) revealed a significantly reduced number of plaques (Figure 2B) and significantly lower mean lesion areas (Figure 2C) in comparison to WD-fed littermate ApoE^−/−^/CRP4^+/+^. This apparently proatherogenic effect was mainly seen in atheroprone regions of the aortic arch (Supplementary Figure S2B,C) and was not associated with changes in the blood lipids in CRP4^−/−^ under WD. Accordingly, the total cholesterol, LDL and very low-density lipoproteins (VLDL) increased to similar levels in both genotypes, and all values determined in WD-fed mice were massively higher than under CD (Figure 2D and Supplementary Table S1), which did not develop vascular lesions after a 16-week feeding period (Figure 2A and Supplementary Figure S2C). Further, the liver weight, total body weight (Supplementary Table S1) as well as the rate of weight gain at each time point (data not shown) were amplified in ApoE^−/−^/CRP4^+/+^ and dKO mice receiving WD with no differences between genotypes (Supplementary Table S1), suggesting that CRP4 does not affect body weight under different dietary conditions. To validate the Oil Red O-based quantifications (Figure 2A), the volume and structure of atherosclerotic lesions were examined in more detail in H&E-stained cross sections (Figure 2E). To assess the progression of atherosclerotic lesions, the media thickness (data not shown), media thickness beneath the plaques and intima-to-media ratio (IMR) were measured (Figure 2F,G). The wall thicknesses in atherosclerotic vessels were not altered between genotypes, while the IMR was significantly enhanced in ApoE^−/−^/CRP4^+/+^ aorta, confirming favourable effects on the extent of atherosclerosis when CRP4 is lacking as observed in dKO (Figure 2G).

Our previous studies indicated that the global lack of CRP4 (on an ApoE^+/+^ background) leads to hypotension [27,29] and blood pressure (BP) is an important determining factor for plaque formation. Thus, we re-measured BP in CRP4-proficent versus -deficient mice concurrently lacking ApoE to evaluate whether hemodynamic effects contributed to the anti-atherogenic phenotype seen in dKO mice. The systolic, diastolic BP as well as the mean arterial pressure did not differ significantly between genotypes upon 16 weeks of WD feeding (Supplementary Figure S3 and Supplementary Table S1). Interestingly, diastolic values were slightly but significantly lower in dKO mice receiving the WD for 12 weeks (data not shown). This implicates an age- and/or diet-related normalization in BP. Owing to these progressively converging values in ApoE^−/−^/CRP4^+/+^ and dKO mice, we conclude that BP alone does not explain the pro-atherogenic effects of CRP4.

### 3.3. Loss of CRP4 Results in a Reduced α-SMA Content in Atherosclerotic Vessels and in Lower Migratory Capacities of Primary VSMCs

**Figure 3:**
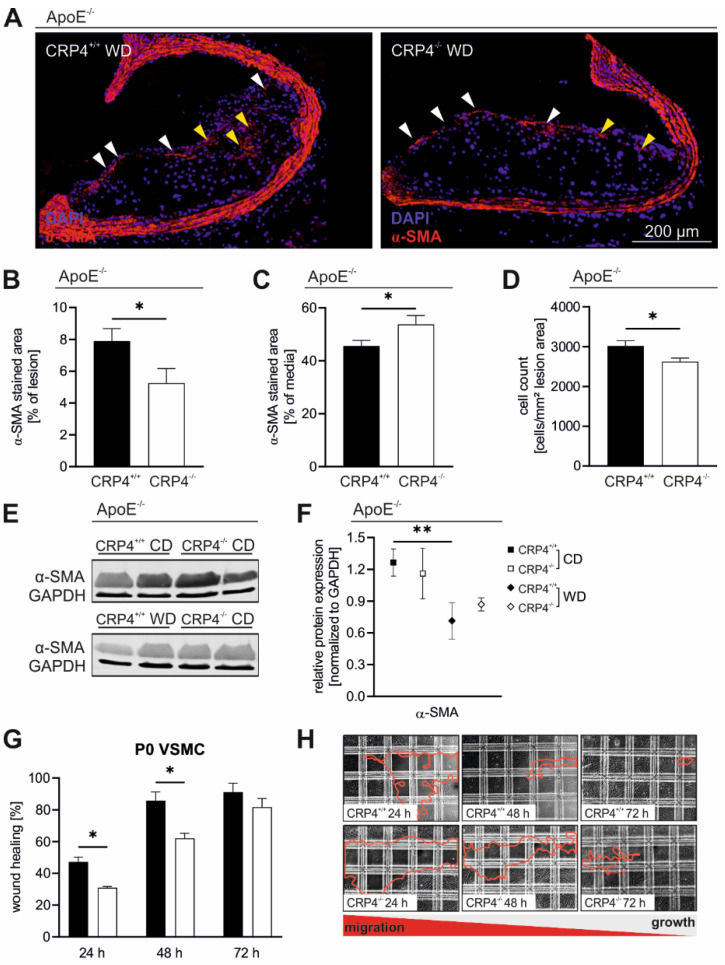
*Lower α-SMA levels in CRP4-deficient atherosclerotic lesions*. (**A**) Immunofluorescence staining using α-SMA-specific antibodies (red) and Hoechst (blue) of atherosclerotic plaques from ApoE^−/−^/CRP4^+/+^ versus ApoE^−/−^/CRP4^−/−^ mice. (**B**) In the absence of CRP4, α-SMA-abundance in the lesion (yellow arrowheads) and cap (white arrowheads) area was significantly decreased, while (**C**) in the media, the α-SMA expression was exactly the opposite in CRP-proficient versus -deficient vessels. (**D**) The number of total cells was estimated by counting the intra-plaque nuclei. The number of nuclei was lower in the absence of CRP4; *n* = 8–9 per genotype, * *p* < 0.05 and two-tailed Student’s *t*-test. (**E**,**F**) Western blot analyses confirmed the decrease of α-SMA in the aorta of WD-fed versus CD-fed groups, although no genotype-specific difference was apparent. GAPDH was used as the loading control for the blots, at *n* = 4 per genotype with each *n* representing a pool of two aorta. The red line in (**H**) highlights the area that remained uncovered by the VSMCs at the time points indicated; ** *p* < 0.01 and two-tailed Student’s *t*-test. (**G**) P0 VSMCs (P0), resembling cells that initiated the phenotype switch, showed a lower migrative behaviour in vitro in the absence of CRP4 compared with CRP4 WT cells; *n* = 4 per genotype with each *n* representing a pool of two aorta; * *p* < 0.05 and two-tailed Student’s *t*-test. Data presented in (**B**–**D**,**F**,**G**) are expressed as the mean ± SEM.

The vascular α-SMA expression pattern was investigated to estimate the contribution of VSMC-like cells to vascular lesion formation [48]. Although this view is not without controversy, previous reports suggested that only a few α-SMA positive medial VSMCs migrate into the intima to support plaque formation by clonal expansion [49,50]. α-SMA positive lesion areas were significantly increased in ApoE^−/−^/CRP4^+/+^ mice, while in the tunica media and the media layer beneath the plaque, the α-SMA staining pattern was significantly broader in the absence of CRP4 (Figure 3A–C). The high prevalence of intra-plaque α-SMA in ApoE^−/−^/CRP4^+/+^ correlated with a higher lesion cell count (Figure 3D) and smaller necrotic core (NC) areas (Supplementary Figure S4). As an explanation for this finding, we speculated that CRP4-positive VSMCs from the media invaded the lesion area resulting in intra-plaque α-SMA-positive cell accumulation (Figure 3D).

α-SMA protein expression was attenuated by WD compared to CD in ApoE^−/−^/CRP4^+/+^ and ApoE^−/−^/CRP4^−/−^ mice in aortic protein lysates (Figure 3E,F). WD downregulated α-SMA in both genotypes as compared to CD, a finding that agrees with previous work [2,8,18,51]. Given the fact that plaque cell proteins represent only a small fraction of the total aortic protein lysates (Figure 2C), it is not surprising that the immunoblot-based analysis did not recapitulate the genotype-specific differences seen in α-SMA stained ApoE^−/−^/CRP4^+/+^ versus dKO plaques (Figure 3B).

The higher α-SMA abundance in the lesion area of atheroprone ApoE^−/−^/CRP4^+/+^ vessels prompted us to evaluate the migratory capabilities of primary unpassaged (P0) VSMCs in culture as surrogates. VSMC cultures were established from multiple young CRP4 WT and KO animals, and migration was monitored using a modified wound healing assay with a removable silicone insert, which ensures uniform gap formation between confluent cell layers [52]. Gap closure was monitored for 24 to 72 h and was attained significantly faster in the CRP4 WT genotype (Figure 3G,H). Significant differences in cell migration into the gap were only observed during earlier time points, i.e., at 24 and 48 h suggesting that CRP4 exhibits a pro-migratory function in P0 VSMCs, while the progressive closure of the gap as well as proliferative cell behaviours abolished these differences at 72 h. In agreement with the increased plaque size (Figure 2C,G), intra-plaque cell counts (Figure 3D) and the α-SMA expression pattern (Figure 3A,B), these findings provide strong evidence that CRP4 promotes the initial switch of the VSMC phenotype in vivo and in culture. This is also consistent with the idea that the first hours and days of cultivation activate phenotypic VSMC changes and thereby the adhesive and migratory capabilities of these cells [4,38,53], while at later time points, the proliferative capacity of phenotypically switched VSMCs potentially masks cell-migration-induced differences in wound healing assays [54].

### 3.4. Elevated Proliferation Rates in Synthetic VSMCs In Vitro and in Atherosclerotic Plaque Cells Lacking CRP4

**Figure 4:**
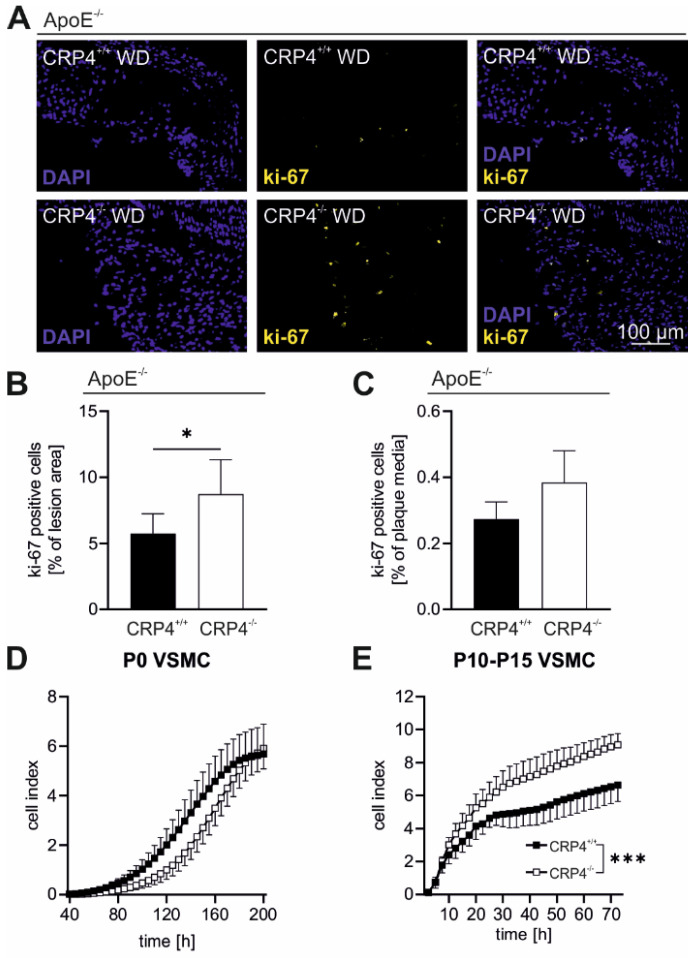
*The ki-67 content of atherosclerotic lesions is dependent on the plaque’s CRP4 status*; (**A**,**B**) Ki-67(yellow) and Hoechst (blue) stained ApoE^−/−^/CRP4^+/+^ and dKO aortic cryosections showed a significantly higher percentage of ki-67-stained cells in the lesion area lacking CRP4, while (**C**) ki-67-positive nuclei did not differ in the media; *n* = 6–7 per genotype, * *p* < 0.05 and two-tailed Student’s *t*-test. (**D**) Impedance-based measurements in real-time of P0 VSMCs revealed a tendentially accelerated growth behaviour in CRP4 WT cell, while in contrast (**E**) in P10-15 cells CRP4 KO VSMCs exhibited higher proliferative behaviour; *n* = 3 per group with each *n* representing a pool of four aorta, *** *p* < 0.001 and two-way ANOVA test. All data expressed in panel (**A**–**E**) are the mean ± SEM.

Proliferation of VSMCs quantitatively contributes to plaque cell accumulation in atherosclerosis [50]. ApoE^−/−^/CRP4^−/−^ displayed a small but significant increase in the proliferation marker ki-67 after 16 weeks of WD (Figure 4A,B). In the medial layer beneath the plaque, the number of proliferating cells was several-fold lower and not different between genotypes (Figure 4C). This finding was unexpected since plaque areas from CRP4-deficient mice were smaller (Figure 2C) also with respect to the IMR (Figure 2G). However, the NC areas of dKO lesions were larger (Supplementary Figure S4) and atherosclerotic lesions contained multiple proliferative cell types, including highly synthetic VSMCs, which may even lose their contractile marker α-SMA (Figure 3B). To examine whether VSMC CRP4 contributes to the proliferative behaviours of this cell type, we conducted an impedance-based real-time growth assay with either P0 VSMCs or highly synthetic VSMCs that were kept in culture for 10 to 15 passages (P10-15). Analysis of P0 VSMCs, having not yet switched to the synthetic phenotype, revealed no genotype-specific difference in the time-dependent increase of the cell index (CI) (Figure 4D). In contrast, in P10-15 VSMCs exhibiting synthetic cells that grew much faster than P0 VSMCs, the lack of CRP4 significantly accelerated the CI in comparison to WT (Figure 4E). In early atherosclerotic stages, CRP4 promotes cell motility (Figure 3G), while the impedance-based proliferation assay implies that CRP4 exhibits anti-proliferative effects in synthetic VSMCs (Figure 4E). The latter finding might explain the increased ki-67 of dKO plaques (Figure 4A) as many cells in atherosclerotic lesions are synthetic VSMCs. Interestingly, such genotype-specific differences in intra-plaque cell proliferation were not observed at earlier stages of atherosclerosis, i.e., after 8 weeks of WD (Supplementary Figure S5A–C). This may suggest that anti-proliferative features of CRP4 become relevant in advanced vascular lesions with P10-15 VSMCs resembling the intra-plaque cell type in advanced lesions upon 16 weeks of WD.

In conclusion, a higher total plaque burden of ApoE^−/−^/CRP4^+/+^ in vivo seems to result from a prominent pro-migratory behaviour of VSMCs (Figure 2), while anti-proliferative effects exhibited by CRP4 (Figure 4B,E) are apparently restricted to synthetic VSMCs in plaques.

### 3.5. Loss of CRP4 Causes Significant Alterations in the VSMC Proteome and has Adverse Effects on Oxidative Stress and Vascular Calcification

To provide insights into the homeostatic regulation of VSMC plasticity by CRP4 we performed LC-MS/MS-based proteomic analysis utilizing the total protein lysates derived from highly passaged P10-15 CRP4 WT and CRP4 KO VSMCs as surrogates for advanced intra-plaque VSM-like cells. This approach identified multiple significantly up- or downregulated proteins, which have either been previously associated with atherosclerosis [55,56,57] or were considered interesting candidates due to their specific function in VSMCs (Table 1).

To identify common biological processes of the VSMC proteins that were significantly altered in abundance by CRP4 deficiency, these proteins were subjected to gene enrichment analysis using GetGo [46]. This resulted in the identification of a single biological process, namely “oxidation reduction” (GO: 0055114). We found that 4 of the 13 proteins that showed an alteration in abundance were involved in this process (*Acadl, Mical2, Pir* and *Prdx4*). Among the proteins upregulated in the absence of CRP4, we identified peroxiredoxin 4 (PRDX4), a protein with thioredoxin-dependent peroxidase activity, which reduces peroxides and other reactive oxygen species (ROS) [73], acyl-CoA dehydrogenase long chain (ACADL) [58] and microtubule-associated monooxygenase (MICAL2), all three being part of the cell’s antioxidant defence mechanism, while Pirin (PIR), a redox sensor for NF-кB [62], was downregulated in highly synthetic VSMCs lacking CRP4.

**Figure 5:**
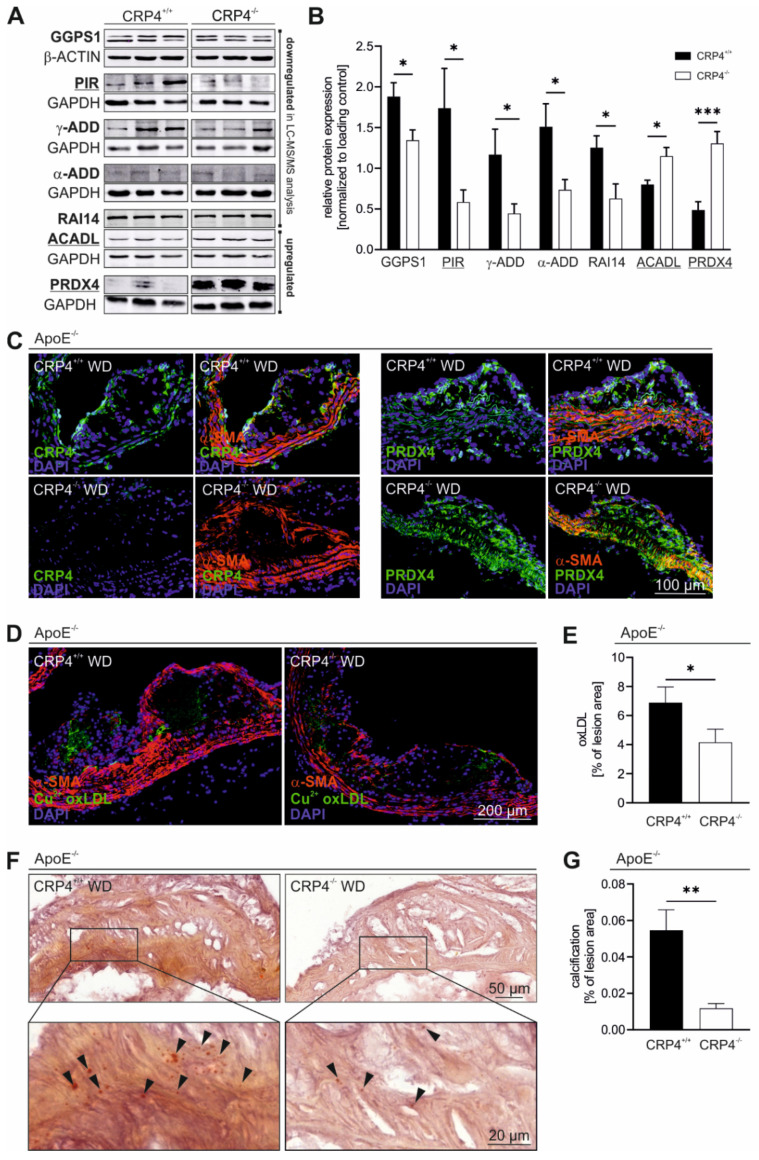
*Vascular CRP4 regulates the expression of several proteins playing key roles in “oxidation reduction” and in atherosclerotic plaque formation*. (**A**,**B**) Significantly regulated proteins identified in LC-MS/MS proteomic analysis (Table 1) of CRP4 WT and KO synthetic VSMCs were validated applying immunoblot technique. GetGo analysis indicated an enrichment of proteins involved in “oxidation reduction” (GO: 0055114) (underlined). Geranylgeranyl diphosphate synthase 1 (GGPS1), retinoic acid induced protein (RAI) 14, alpha- and gamma-Adducin (α- and γ-ADD) and Pirin (PIR) were significantly depleted in protein lysates obtained from P10-15 VSMCs lacking CRP4, whereas peroxiredoxin (PRDX) 4 and acyl-CoA dehydrogenase long chain (ACADL) were confirmed as significantly upregulated proteins in CRP4 KO, GAPDH and β-actin were co-detected to demonstrate equal loading of the gels; *n* = 7–11 per genotype where *n* represents a pool of four aorta, * *p* < 0.05, *** *p* < 0.001 and two-tailed Student’s *t*-test. (**C**) Immunofluorescence-based co-detection of CRP4 (green) and PRDX4 (green) as well as α-SMA (red) in frozen aortic tissue sections. CRP4 (left panel) was present in media and in intra-plaque cells, and it was detected in the fibrous cap of CRP4^+/+^ but not CRP4^−/−^. PRDX4 (right panels) was expressed in medial and plaque cells, and it was significantly upregulated in dKO atherosclerotic plaques and the underlying media. Intra-plaque PRDX4 did not overlap with α-SMA suggesting that these cells derived from a non-VSMC source or that these cells resemble VSM-like cells that lost α-SMA during dedifferentiation; *n* = 4 to 5 for the CRP4 and PRDX4 staining per genotype. (**D**,**E**) OxLDL was visualized using a Cu^2+^ oxLDL antibody (green). A lack of CRP4 was associated with a significant lower oxLDL content in atherosclerotic plaques. Co-staining with α-SMA (red) was performed to better clarify the localization of oxLDL within the different plaque structures; *n* = 6–7 per genotype, * *p* < 0.05 and two-tailed Student’s *t*-test. (**F**,**G**) Alizarin staining of aortic cryosections identified a potentially protective plaque-calcification defect in dKO compared to ApoE^−/−^/CRP4^+/+^ lesions; *n* = 7 per genotype, ** *p* < 0.01 and two-tailed Student’s *t*-test. Data shown in (**B**,**E**,**G**) are expressed as the mean ± SEM.

Three differentially expressed proteins related to redox signalling as well as four other protein candidates (α- and γ-Adducin (ADD1 and 3), GGPS1 and RAI14) discovered by the proteomic approach were verified by immunoblots utilizing P10-15 VSMC-derived protein lysates (Figure 5A,B). Based on this result, we selected one protein for further validation to demonstrate the relevance of the proteomic approach. We focused on the antioxidant enzyme PRDX4, a protein that was highly upregulated in lysates obtained from CRP4 null lysates, because its overexpression reportedly correlated with cell protective, plaque stabilizing effects in atherosclerosis as well as the attenuated formation of lesions [56,74] and oxidative stress [73,75]. To verify whether PRDX4 is also expressed in the lesion area and/or intra-plaque VSMCs, we co-stained aortic cryosections of ApoE^−/−^/CRP4^+/+^ versus dKO vessels with PRDX4 and α-SMA antibodies (Figure 5C). PRDX4 was unevenly distributed in ApoE^−/−^/CRP4^+/+^ plaques and media. The overlap with α-SMA suggests that VSMCs and/or cells of VSMC origin, in addition to other cell types, express PRDX4 in vivo in the vasculature. In dKO plaques lacking CRP4 (Figure 5C), however, higher levels of PRDX4 were detected in the media beneath the plaque, in cap cells and in α-SMA-negative cells of the plaque core (Figure 5C).

The high abundance of the PRDX4 protein scavenged ROS and affected plaque-related lipid oxidation as indicated by a Cu^2+^-oxLDL-specific antibody (Figure 5D). This antibody detected the oxidized LDL particles [56] in ApoE^−/−^/CRP4^+/+^ plaques. However, the lack of CRP4 significantly reduced the amount of Cu^2+^-oxLDL deposition in dKO (Figure 5D,E).

Since oxidative stress and increase in oxLDL induces osteochondrogenic pathways leading to vascular calcification [76,77,78], calcium deposits in plaques were quantified by Alizarin staining (Figure 5F,G). The extent of calcification in dKO lesions was very low and impressively reduced compared to ApoE^−/−^/CRP4^+/+^ lesions (Figure 5F,G). Both oxLDL and calcification constitute destructive events in advanced lesions and predispose cells to plaque rupture [79]. However, the parameters of plaque vulnerability, such as changes in the stabilizing extracellular matrix (ECM) proteins, elastin fragmentation in the vessel wall and the total intra-plaque amount of lipids did not differ in the presence or absence of CRP4 (Supplementary Figure S6A–F). Importantly, immunoblot and immunofluorescence quantifications of macrophage marker mac2 showed that macrophage-like cells in atherogenic plaques increased in WD versus CD irrespective of the CRP4 genotype (Supplementary Figure S6G–J).

**Figure 6:**
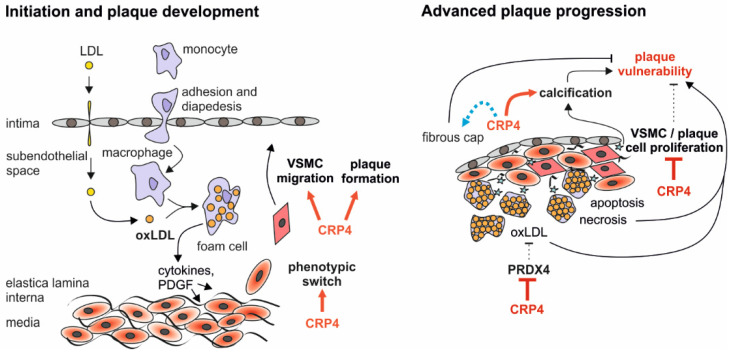
*Synopsis of the putative functions attributed to CRP4 in atherosclerotic plaque initiation and progression.* Proatherogenic CRP4 enhances plaque building by promoting the phenotypic switch and migratory properties of VSMCs. This results in a higher amount of α-SMA expression in the lesion area and an overall higher plaque burden. In advanced plaques, CRP4 may predominantly affect plaque stability through inhibition of the antioxidant enzyme PRDX4. Lower PRDX4 abundance in CRP4 proficient VSMCs and vessels causes a higher level of lesion oxLDL, and this is associated with an enhanced calcification. *Vice versa*, the anti-proliferative effects of CRP4 on VSMC/plaque cell proliferation may promote a vulnerable state of atherosclerotic plaques. However, the NC of ApoE^−/−^/CRP4^+/+^ lesions are smaller. Since no threshold above which a plaque becomes unstable has been determined, the significance of the latter finding remains unclear.

In sum, CRP4 is a novel proatherogenic modifier influencing important determinants of atherosclerotic plaque “in-/stability” (Figure 6).

## 4. Discussion

The data presented herein imply that CRP4 is a novel regulator of atherosclerotic plaque development. Pathological feeding conditions primed genotype-specific differences between the ApoE^−/−^/CRP4^+/+^ and dKO vasculature. In detail, we identified multiple adverse features in ApoE^−/−^/CRP4^+/+^ lesions that maybe attributed to VSMC CRP4: (i) an increase in plaque size and number, (ii) lesional hypercellularity, (iii) discrete changes in the enrichment of proteins involved in “oxidation reduction”, (iv) a rise in oxidation of intra-plaque LDL and (v) higher susceptibility to calcification events in vivo. Although the potential reasons for these pro-atherogenic effects of CRP4 need to be examined in detail in the future, the current evidence supports the notion that the CRP4-proficiency of P0 VSMC associates with an accelerated migratory capacity in vitro as a key underlying factor for plaque formation in ApoE^−/−^/CRP4^+/+^ mice. Indeed, lineage-tracing studies support the notion that atherosclerotic lesions are composed of at least 30% VSMC-derived cells [13,80]. While the initial steps of atheroma formation are stimulated by VSMC CRP4, it should be considered that CRP4 may exhibit opposing roles in synthetic VSMCs. In this cell type, the presence of CRP4 prevented the downregulation of the contractile marker α-SMA, and it attenuated the proliferative capacity of highly synthetic P10-15 VSMCs in vitro. Together with the higher ki-67 status and larger NC areas of advanced lesions lacking CRP4, these findings suggest that CRP4 plays a double-edged role during different stages of VSMC plasticity and atherosclerosis. Considering the milder atherosclerotic phenotype of dKO mice, we conclude that the pro-atherogenic effects of CRP4 dominate over its antiproliferative role in highly synthetic VSMCs, at least in vivo.

As implicated for CRP4, related members of the CRP4 family, such as CRP1 and CRP2, which exhibit regulatory functions on the actin cytoskeleton, have previously been linked to VSMC proliferation [33,34] as well as differentiation [35]. In addition to their effects on the cytoarchitecture, CRP1 and CRP2 form complexes with SRF and GATA transcription factors, facilitating the expression of some SM marker genes [34,35]. Our preliminary findings, however, imply no compensatory regulation of CRP1 and CRP2 in CRP4^−/−^ vessels (data not shown). Accordingly, Zhang and co-workers demonstrated that the adapter protein CRP4, by cooperatively interacting with the transcription factors SRF and GATA6, regulates the expression of VSMC-specific genes in SMC-like cells in vitro [21,23], while the role of CRP4 for the phenotypic switch of VSMCs, its role in primary versus synthetic VSMCs as well as putative disease-relevant aspects of the transcriptional observation remained elusive. We here confirm that CRP4 is expressed mainly in the medial layer of the aorta. At a cellular level, it is highly abundant in the cytoplasm, especially in the perinuclear region of isolated VSMCs from the aorta (Figure 1A–C). In the atheroprone ApoE^−/−^ model, the migration of medial VSMCs into the lesion area is a major contributor of plaque formation and progression [81,82]. Interestingly, unpassaged CRP4 WT VSMCs presented with a higher migration in vitro (Figure 3G,H) leading to an accelerated plaque build-up in vivo (Figure 2A–C,E,G) and thereby to smaller NC areas with a higher α-SMA expression in atherosclerotic lesions (Supplementary Figure S4 and Figure 3A,B).

While CRP4 primes cells more to the migratory phenotype, thereby, accelerating plaque formation and size (Figure 2), its presence supresses cell proliferation in advanced atherosclerotic plaques as well as in synthetic VSMCs in vitro (Figure 4A,B,E). Accordingly, the number of cells expressing the proliferation marker ki-67 was decreased in ApoE^−/−^/CRP4^+/+^ plaques (Figure 4A), while the total plaque cell counts were increased in comparison to dKO mice (Figure 3D), which is in line with a smaller NC area (Supplementary Figure S4). A possible explanation for this paradoxical finding is that the global lack of CRP4 causes changes in non-VSMC-derived plaque cell types and their turnover; albeit, our mac2 analyses (Supplementary Figure S6G–J) argue against any relevant alterations in plaque-associated macrophages between genotypes.

In advanced lesions, higher VSMC proliferation rates are usually considered as one important determinant of plaque stability [2]; however, the different properties of VSMCs and their relevance to different pathological states in atherosclerosis is complex [1,83]. Providing further support for the concept that the plaque’s CRP4 status is associated with the instability comes from the increased deposition of intra-plaque oxLDL particles and micro-calcifications in ApoE^−/−^/CRP4^+/+^ versus dKO plaques (Figure 5D–G). Interestingly, oxidized lipid accumulation in the subendothelial space associates with calcifying activity in VSMCs and arterial calcification, while “native” LDL does not [84]. Moreover, oxLDL and hydrogen peroxide exposure of VSMCs reportedly induced osteogenic transdifferentiation [76,77,78], leading to vascular differentiation. Although our analysis of additional markers of plaque in-/stability did not reveal differences between genotypes (Supplementary Figure S6A–F), these findings suggest that the plaque’s positive CRP4 status predicts rupture-prone plaques—although spontaneous plaque rupture in mice is a controversial topic.

Oxidative stress is involved in a variety of pathophysiological conditions, including inflammation, ischemia and reperfusion injury, obesity and insulin resistance, and it is thought to contribute to lesion formation in atherosclerosis [15,85,86,87]. ROS enhance atherosclerosis by several mechanisms [88,89], including the oxidation of LDL as an important early trigger for this vascular disease [15,86,90]. Moreover, the oxidation of LDL is cytotoxic for all cells involved in atherogenesis. Our analysis of the global protein profile in synthetic VSMCs implied that PRDX4, a peroxidase that catalyses the detoxification of ROS, such as hydrogen peroxide, was massively upregulated by lack of CRP4 (Figure 5A,B). PRDX4-mediated scavenging of ROS has been described for the intracellular endoplasmic reticulum as well as the extracellular lumen [56,74,91,92]. Accordingly, PRDX4 is secreted in inflammation [61,93] and it negatively regulates NF-кB signalling [94,95] in atherosclerosis [96]. Moreover, several members of the Prdx family (e.g., Prdx1, 2 and 6) contribute to antioxidative signalling and thereby to cascades that protect against atherosclerosis-prone and mechanically induced vessel pathophysiology [97,98,99]. Consistently, transgenic overexpression of the human PRDX4 protein in the ApoE-deficient mouse model resulted in lower levels of oxidative stress markers and oxLDL in atherosclerotic plaques [56]. Thus, strategies or agents inducing PRDX4 might be exploited for their potential to suppress oxidative damage and plaque vulnerability in atherosclerotic disease. Interestingly, these antiatherogenic effects of PRDX4 were not linked to changes in glucose and lipid levels, thereby, suggesting direct effects of PRDX4 on plaque pathogenesis. These results are in line with our in-depth analysis of ApoE^−/−^/CRP4^+/+^ and dKO models proving evidence for the important anti-atherogenic potential of PRDX4. An amplified expression of this antioxidant enzyme was observed in highly synthetic VSMCs (Figure 5A,B) in the plaque and in the media beneath the plaque of ApoE^−/−^/CRP4^−/−^ mice (Figure 5C). Low intra-plaque PRDX4 levels, in turn, correlated with high oxLDL deposition suggesting that CRP4, by negatively regulating PRDX4 in plaque-associated VSMCs promotes the oxidative stress of vulnerable lesions. It is currently unclear whether the GATA6- and SRF-dependent regulation of SM-specific gene transcripts by CRP4 [21] plays a role in *Prdx4* expression.

Our analysis of the proteomic profile of synthetic VSMCs identified additional up- or downregulated proteins. In addition to PRDX4, three additional proteins related to the “oxidation reduction” (GO: 0055114) process were identified by the GetGo tool (Table 1). Two of these three proteins (ACADL and PIR) as well as four proteins with different functions (α-/γ-ADD, GGPS1 and RAI14) were verified by immunoblotting confirming the validity and accuracy of our proteomic approach. Interestingly, the majority of significantly regulated proteins (Table 1) have previously been recognized for their role in VSMC differentiation, in organizing the dynamics of the actin cytoskeleton and/or their functional impact on the pathogenesis of atherosclerosis.

Of note, and in addition to PRDX4, ACADL and PIR, microtubule-associated monooxygenase (MICAL2), an atypical actin-regulatory protein, has been implicated in redox-regulated processes in VSMCs [59]. This large, multidomain flavin-dependent monooxygenase induces a posttranslational oxidation of F-actin on methionine 44/47 leading to the disassembly of F-actin as a fundamental unit of the cytoskeleton in the nucleus [60,100]. Although highly speculative, this oxidation-dependent mechanism regulating actin dynamics provides a logic whereby the high abundance of MICAL2 in CRP4 null VSMCs may result in reduced migratory cell behaviour.

Even though we did not study whether the well-established connection between the cGMP/cGKI signalling pathway and CRP4 in SMC-like cells in vitro has any implication on the atherosclerosis development in vivo, this topic deserves some attention. For example, the NO-sensitive guanylyl cyclase (NO-GC), a major source for vascular cGMP production, promoted proliferative and migratory behaviours of VSMCs and, thereby, the formation of atherosclerosis [40]. Along these lines, a lack of VSMC cGKI, the major downstream effector of cGMP in VSMCs and many other cell types, reduced plaque size in the ApoE^−/−^ mouse model [38]. Although opposing views exist [41], this evidence deriving from two genetically targeted mouse models lacking either the α_1_ subunit of the NO-GC heterodimer or cGKI strongly support a pro-atherogenic role for the endogenous NO-GC/cGMP/cGKI pathway, which is consistent with our present report on the pathophysiological functions of CRP4 in VSMC plasticity and atherosclerosis development. However, the cGKI substrate CRP4 may also act as a negative feedback regulator on the spatial activity of the cGMP axis on vascular tone and BP regulation [29]. Given the pro- and anti-atherosclerotic effects of cGMP on the vessel wall, the putative link to CRP4 must await future studies of atheroprone models.

In conclusion, we identify a proatherogenic role of the protein–protein interactor CRP4 assessing a global CRP4 WT versus KO model lacking ApoE. Plaque building, by a pro-migratory function of CRP4 during early steps of the phenotypic VSMC switch, was accelerated in vivo leading to higher amount of α-SMA positive cells in atherosclerotic lesions. The dysregulation of several proteins (ACADL, PIR and MICAL2) related to “oxidation reduction” processes, such as the antioxidant enzyme PRDX4 in synthetic VSMCs and in aortic plaques was identified as a crucial determinant of the anti-atherogenic phenotype of dKO mice. Vascular calcification and oxLDL, but not total lipid deposition, were more frequently observed in CRP4-proficient lesions. These findings suggest that CRP4 stimulates multiple adverse properties of VSMC phenotypic modulation (Figure 6).

Strategies to carefully modulate CRP4 should attenuate the migratory potential of VSMCs and induce PRDX4. This may prevent early lesion formation to improve plaque stability at later stages, whereas the concomitant increase in NC areas may be an undesired side effect of such interventions.

## 5. Limitations of the Study

Last, our findings engender new questions, as other cell types, such as endothelial cells, platelets, and multiple types of immune cells, may also contribute to the in vivo phenotype of the global dKO mice analysed in this study. This will require (inducible) cell-specific approaches to address the contribution of CRP4 in non-VSMC cell compartments to atherogenesis in vivo. However, pre-mutant *Crip2* models that would allow for a conditional ablation of CRP4 by Cre/loxP are presently not available, which is a major shortcoming of our study.

In addition to the switching of mature to synthetic VSMCs in atheromatous vessels, VSMCs undergo apoptosis and senescence, and they are also capable of transdifferentiating into alternative cell types, such as foam cells, macrophages, mesenchymal stem cells, osteochondrogenic cells and ECM-producing cells [3]. These multiple features of VSMCs contribute to the progression of atherosclerotic lesions, intra-plaque inflammation, oxidative stress as well as cell death as important determinants of plaque rupture [101]. Although pro- and anti-atherogenic roles of VSMCs in atheromatous vessels bear huge potential for future treatments, currently these approaches are still at the very beginning [3]. The potential of CRP4 to contribute to any of these processes must be addressed by sophisticated lineage-tracking studies in atherosclerosis-prone mice [13]. To pinpoint the mechanisms underlying atheroma formation by CRP4, these future analyses should consider the evaluation of MMPs, inflammatory cytokines and an in-depth assessment of the redox signalling processes in VSMCs. Based on our proteomic approach, the promising candidates that may contribute to vessel injury and/or VSMC phenotype modulation in a CRP4-dependent manner are ACADL, PIR, and MICAL2 in addition to PRDX4.

## Figures and Tables

**Table 1:** Proteomic analysis of P10-15 VSMCs.

Protein	Gene Name	*t*-Test *p*-Value	Significance *B* Value	Regulation in CRP4^−/−^	Selected Reference(s)
**Oxidation Reduction Related Pathways GO: 0055114**					
Acyl-CoA dehydrogenase long chain (**ACADL**)	*Acadl*	0.027	0.004	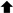	[58]
Microtubule associated monooxygenase, Calponin and LIM domain containing 2 (**MICAL2**)	*Mical2*	0.025	0.002	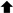	[59,60]
Peroxiredoxin-4 (**PRDX4**)	*Prdx4*	0.016	0.001	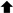	[56,61]
Pirin (**PIR**)	*Pir*	0.012	0.000	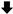	[62]
**Other pathways**					
α-Adducin (**α-ADD**)	*Add1*	0.045	1.01 × 10^−6^	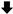	[63,64]
Ankycorbin (**RAI14**)	*Rai14*	0.029	3.5 × 10^−4^	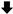	[65]
Basal cell adhesion molecule (**BCAM**)	*Bcam*	0.012	1.11 × 10^−320^	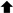	[66]
γ-Adducin (**γ-ADD**)	*Add3*	0.024	1.01 × 10^−8^	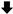	[64]
Geranylgeranyl diphosphate synthase 1 (**GGPS1**)	*Ggps1*	3.9 × 10^−12^	3.12 × 10^−73^	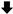	[67,68]
Interferon-induced transmembrane protein 3 (**IFITM3**)	*Ifitm3*	0.038	0.002	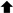	[69,70]
Platelet activating factor acetylhydrolase 1b catalytic subunit 3 (**PAFAH1B3**)	*Pafah1b3*	0.021	0.004	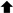	[57]
THO complex subunit 2 (**THOC2**)	*Thoc2*	0.008	1.58 × 10^−61^	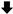	[71]
Ubiquitin like modifier activating enzyme 1 (**UBA1**)	*Uba1*	0.010	4.57 × 10^−5^	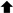	[55,72]

Liquid chromatography–tandem mass spectrometry (LC-MS/MS) analysis of synthetic VSMCs (P10-15) identified several proteins that were either up- or down-regulated in a CRP4-dependent manner as indicated by the arrows. Gene Ontology enrichment analysis using the relative abundance of each protein was calculated from *n* = 5 independent samples per genotype, whereby each sample comprised a pool of *n* = 4 aorta. Quantification and identification were performed with the MaxQuant Software v2.1.0.0 (https://www.maxquant.org/, last accessed on 2 April 2022 and provided by Max-Planck Institute of Biochemistry, Martinsried, Germany). Ratio calculations and statistics were performed with the Perseus software. All presented proteins were identified as significantly regulated proteins in at least one statistical test (paired *t*-test and significance *B* test). The statistical significance is presented as *p*-values.

## Data Availability

The raw data and the analytic methods will be made available to other researchers for purposes of reproducing the results in their own laboratories on reasonable request. To access protocols or datasets contact robert.lukowski@uni-tuebingen.de.

## Supplementary Materials

The following supporting information can be downloaded at: https://www.mdpi.com/article/10.3390/cells11081364/s1, Figure S1: Experimental design of the in vivo atherosclerotic mouse model; Figure S2: En face oil red O staining of ApoE-deficient aorta; Figure S3: Telemetric blood pressure in ApoE^−/−^/CRP4^−/−^ double mutants; Figure S4: Necrotic core areas in ApoE^−/−^/CRP4^+/+^ and dKO lesions; Figure S5: Proliferation rate in ApoE^−/−^ atherosclerotic plaques after 8 weeks of WD; Figure S6: Multiple plaque vulnerability parameters are not affected by CRP4; Table S1. Plasma analysis of metabolic parameters and hemodynamic analysis.
