# Supplementary material for: Cysteine-Rich LIM-Only Protein 4 (CRP4) Promotes Atherogenesis in the ApoE−/− Mouse Model
Source: Cells. 2022 Apr 17;11(8):1364. doi: 10.3390/cells11081364 (PMC9032522; doi:10.3390/cells11081364)
Supplement: Supplementary file 1 [file cells-11-01364-s001.zip › cells-1583018-supplementary.pdf]

Cysteine-rich LIM-only protein 4 (CRP4) contributes to atherogenesis in the ApoE$^{-/-}$ mouse model

Natalie Längst[1], Julia Adler[1], Anna Kuret[1], Andreas Peter[2], Peter Ruth[1], Karsten Boldt[3], and Robert Lukowski[1,*]

[1]*Experimental Pharmacology, Department of Pharmacology, Toxicology and Clinical Pharmacy, Institute of Pharmacy, University of Tübingen, 72076 Tübingen, Germany*

[2]*Department for Diagnostic Laboratory Medicine, Institute for Clinical Chemistry and Pathobiochemistry, University Hospital Tübingen, 72076 Tübingen, Germany*

[3]*Molecular Biology of Retinal Degenerations, Institute of Ophthalmic Research, University of Tübingen, 72076 Tübingen, Germany*

**Correspondence: robert.lukowski@uni-tuebingen.de; Tel.: +49-7071-29-74550, Fax: +49-7071-29-2476.*

Supplemental Information:

Supplemental methods
Supplemental table and Legend (1)
Supplemental Figures and Legends (1-6)
Supplemental References (1)

Supplement Methods

Telemetric blood pressure measurements

The Data Science International telemetry acquisition system (DSI, St. Paul, MN, USA) with TA11PA-C10 transmitters was used for the measurement of basal blood pressure in ApoE$^{-/-}$/CRP4$^{+/+}$ and ApoE$^{-/-}$/CRP4$^{-/-}$ mice after 16 weeks of WD. The implantation of the transmitter was carried out after 12 weeks of WD and was previously described [27]. In brief, the animals received ketamine (80 mg/kg body weight) and xylazine (10 mg/kg body weight) for analgesia. Afterwards anaesthesia was induced by an isoflurane-oxygen mixture (0.5-2%) and the mouse was placed on a heat plate (37°C). Next, a small ventral incision was made submandibular, and the left common carotid artery was cleared from surrounding tissue without disturbing the nervus vagus. The first permanent ligature was set caudal to the bifurcation, followed by a second non-permanent ligature proximal to the first ligature. For the insertion of the catheter the vessel was carefully scribed with a cannula. Then, the catheter was introduced into the left common carotid artery and advanced until its front tip reached the aortic arch. The catheter was secured by tightening the non-permanent ligature and for optimal fixation another permanent ligation was set around the catheter and the vessel approximately 4 mm distal to the first one. The transmitter was positioned subcutaneous in the right flank area of the animal. After week 16 of the WD feeding the blood pressure measurement was performed on 3 consecutive days and the mean of these data was used for statistical analyses.

Oil red O staining

For the visualisation of neutral lipids oil red O staining was performed on aortic cryosections of ApoE$^{-/-}$/CRP4$^{+/+}$ and ApoE$^{-/-}$/CRP4$^{-/-}$ mice after 16 weeks WD feeding was performed. After tissue fixation with 4% PFA in PBS for 10 min the cryosections were incubated in 60% isopropanol for 5 min. Subsequently 10 min oil red O staining (0.5 g Oil red O dissolved in 100 ml isopropanol and diluted with 67 ml ddH_2O) was performed followed by three washing steps with 60% isopropanol each for 2 sec. Then cell nuclei were counterstained with haematoxylin for 6 min. Afterwards the cryosections were washed with tap water several times and were mounted with the aqueous mounting medium Aquatex. The stained cryosections were detected with the Panoramic Desc 3D Histech (Sysmex) and analysed via ImageJ.

Masson trichrome staining

A Masson trichrome staining protocol was applied to visualize collagen fibres in aortic cryosections obtained from ApoE$^{-/-}$/CRP4$^{+/+}$ and ApoE$^{-/-}$/CRP4$^{-/-}$ mice after 16 weeks of WD. First the cryosections were fixed for 24 h in Bouin's solution and washed 3x with tap water. Then, cell nuclei were stained for 1 min in Weigert's iron haematoxylin and washed again for 3 times in tap water. Afterwards the cytoplasm was counter-stained by incubating the sections in Biebrich scarlet-acid fuchsin solution for 3 min. After washing for 3 times in dH_2O the pre-treatment of collagen fibres with phosphomolybdic acid/phosphotungstic acid solution was performed for 5 min. Subsequently anilin blue solution was applied for 2 min and for a better differentiation the cryosections were subsequently incubated for 2 min in acetic acid (1%). Afterwards 4 washing steps in dH_2O followed and dehydration of the stained tissue was carried out with 80% and 100% ethanol followed by 5 min xylene incubation. The non-aqueous medium DPX (Merck Millipore, Darmstadt, Germany) was used for mounting. The stained cryosections were scanned with the Panoramic Desc 3D Histech (sysmex) and analysed with the software ImageJ.

Elastica van Gieson staining

Elastin breaks in aortic cryosections of CRP4$^{+/+}$ and CRP4$^{-/-}$ mice with an ApoE$^{-/-}$ background after 16 weeks of WD were detected with the Elastika van Gieson staining. After 24 h fixation of the cryosections in Bouin's solution two washing steps in tap water were performed. Afterwards, resorcin fuchsin solution was applied for 35 min. Excessive staining solution was removed by 4 washing steps with tap water and subsequently the cell nuclei were stained with Weigert's iron haematoxylin solution for 2 min. Two washing steps in dH_2O and 5 washing steps in tap water followed before the cryosections were incubated in van Gieson solution for 1 min. Differentiation and rehydration of the stained tissue was performed with ethanol in increasing concentrations (70%, 96% and 100%) and 5 min in xylene. The cryosections were mounted with the non-aqueous mounting medium DPX (Merck Millipore, Darmstadt, Germany). Detection of the stained tissue was performed with the Panoramic Desc 3D Histech (Sysmex) and were analysed with the software ImageJ.

Supplement tables and figures

Parameter	ApoE$^{-/-}$/CRP4$^{+/+}$ CD	ApoE$^{-/-}$/CRP4$^{-/-}$ CD	ApoE$^{-/-}$/CRP4$^{+/+}$ WD	ApoE$^{-/-}$/CRP4$^{-/-}$ WD
**Body weight** (g)	29.48 ± 0.68	28.41 ± 1.01	32.91 ± 1.31***	33.42 ± 1.64***
**Liver weight** (mg/mm tibia)	73.78 ± 4.31	74.61 ± 5.54	126.71 ± 17.36**	109.37 ± 9.46*
**HDL** (mg/dL)	29.99 ± 2.03	30.27 ± 2.57	26.09 ± 1.78	25.22 ± 1.79
**LDL** (mg/dL)	25.98 ± 3.03	28.47 ± 2.26	277.60 ± 27.98***	277.43 ± 20.41***
**VLDL** (mg/dL)	358.27 ± 27.12	399.98 ± 26.93	1248.44 ± 103.42***	1324.08 ± 99.38***
**Triglyceride** (mg/dL)	220.41 ± 27.27	189.48 ± 19.21	264.26 ± 35.59	228.80 ± 29.93
**Glucose** (mg/dL)	198.87 ± 16.79	209.88 ± 13.62	245.22 ± 21.02	234.34 ± 15.32
**SBP** (mmHg)	ND	ND	116.99 ± 3.18	112.12 ± 3.07
**DBP** (mmHg)	ND	ND	92.96 ± 2.41	87.14 ± 2.74
**MAP** (mmHg)	ND	ND	105.41 ± 2.58	99.97 ± 2.68
**Pulse pressure** (mmHg)	ND	ND	24.00 ± 2.34	24.66 ± 1.86
**Heart rate** (bpm)	ND	ND	573.96 ± 16.64	534.75 ± 11.97
**Activity** (events/min)	ND	ND	4.78 ± 0.65	6.91 ± 0.86

Supplement Table S1. *Plasma analysis of metabolic parameters and hemodynamic analysis.*

Quantification of body weight, liver weight, blood pressure values and lipid panel (performed on terminal blood drawn via cardiac puncture of 16 weeks WD or CD fed mice as indicated), n=7-15 per group. All data expressed as means ± SEM with * $p < 0.05$, ** $p < 0.01$, *** $p < 0.001$, two-tailed student t-test. *Abbreviations used: HDL, high density lipoprotein; LDL, low density lipoprotein; VLDL, very low density lipoprotein; SBP, systolic blood pressure; DBP, diastolic blood pressure and MAP, mean arterial pressure.*

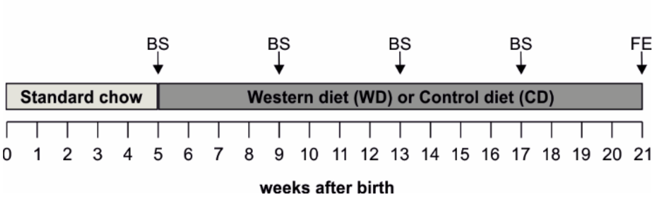

Supplement Figure S1. *Experimental design of the in vivo atherosclerotic mouse model.*

Experimental design for in vivo atherosclerosis studies in $ApoE^{-/-}$ mice. 5 weeks $ApoE^{-/-}/CRP4^{+/+}$ and $ApoE^{-/-}/CRP4^{-/-}$ mice received either a Western (WD) or Control diet (CD) for 8 to 16 weeks. Blood samples (BS) were taken every fourth week and a final blood sample was taken after 16 weeks at the final examination (FE). Tissue harvested at this time point was processed as described for *en face* oil red O staining, IF- and IHC-staining and WB analyses.

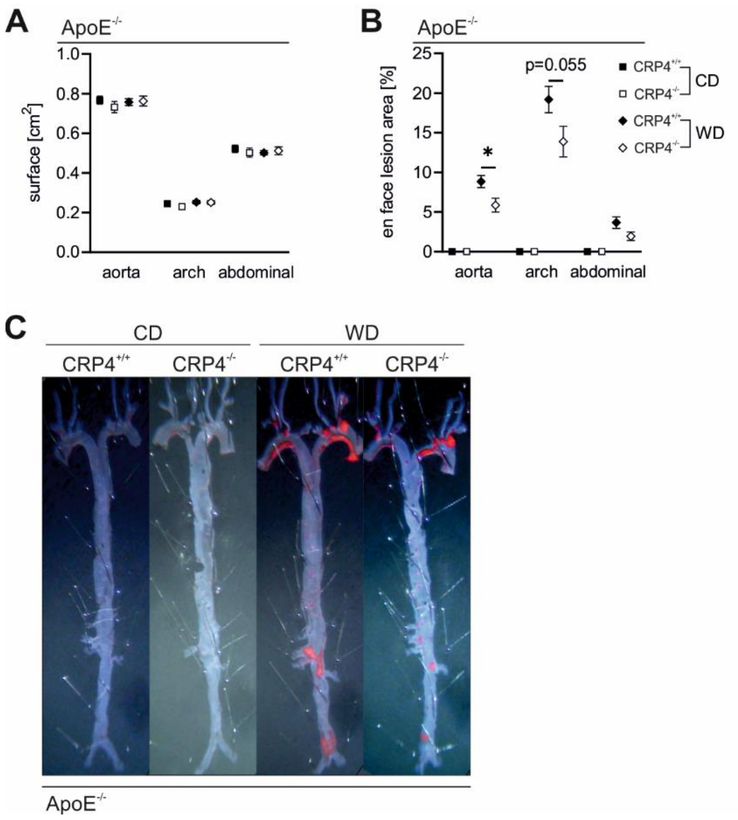

Supplement Figure S2. *En face oil red O staining of ApoE-deficient aorta.*

(**A**) Overall quantification of surface area of *en face* pinned ApoE$^{-/-}$/CRP4$^{+/+}$ and dKO aorta did not differ between the different feeding conditions. (**B**) *En face* lesion area of ApoE$^{-/-}$ mice demonstrated significant higher atherosclerotic lesion areas in CRP4$^{+/+}$ aorta than in CRP4$^{-/-}$. This was due to the higher plaque burden in the abdominal region of dKO vessels, n=12-14 per genotype, * $p < 0.05$, two-tailed student t-test. All data expressed as means ± SEM. (**C**) Representative oil red O staining revealed no spontaneous occurrence of lesions under CD, while atherosclerotic plaque formation was seen in the region of the aortic arch, the abdominal branches, and the iliacal bifurcation under WD.

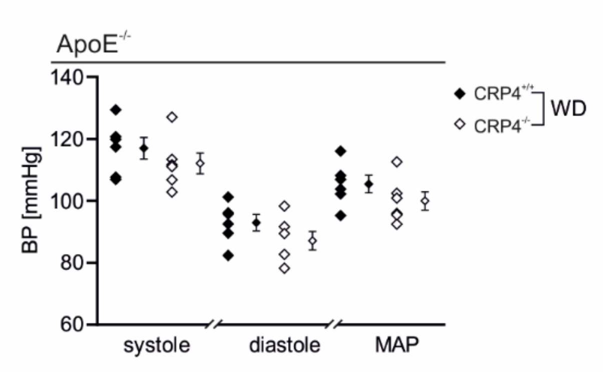

Supplement Figure S3. *Telemetric blood pressure in ApoE$^{-/-}$/CRP4$^{-/-}$ double mutants.*

In vivo blood pressure measurements were performed after 16 weeks of WD in freely moving animals using telemetry. Systolic, diastolic, and mean arterial pressure (MAP) values did not differ between CRP4$^{+/+}$ and CRP4-deficient mice lacking ApoE. All data were expressed as means ± SEM with n=6 per genotype, two-tailed student t-test.

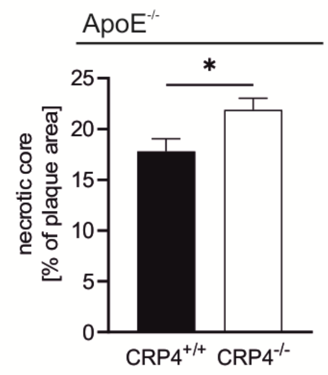

Supplement Figure S4. *Necrotic core areas in ApoE$^{-/-}$/CRP4$^{+/+}$ and dKO lesions.*

Intraplaque necrotic core (NC) areas were determined in cross sections obtained from ApoE$^{-/-}$/CRP4$^{+/+}$ and dKO mice upon WD feeding and demonstrated significant larger NC areas due to CRP4 ablation. NC area was determined as percentual area of the lesions in n=6-7 animals per genotype assessing n=6 cross sections per mouse. All data expressed as means ± SEM with * $p < 0.05$ and two-tailed student t-test.

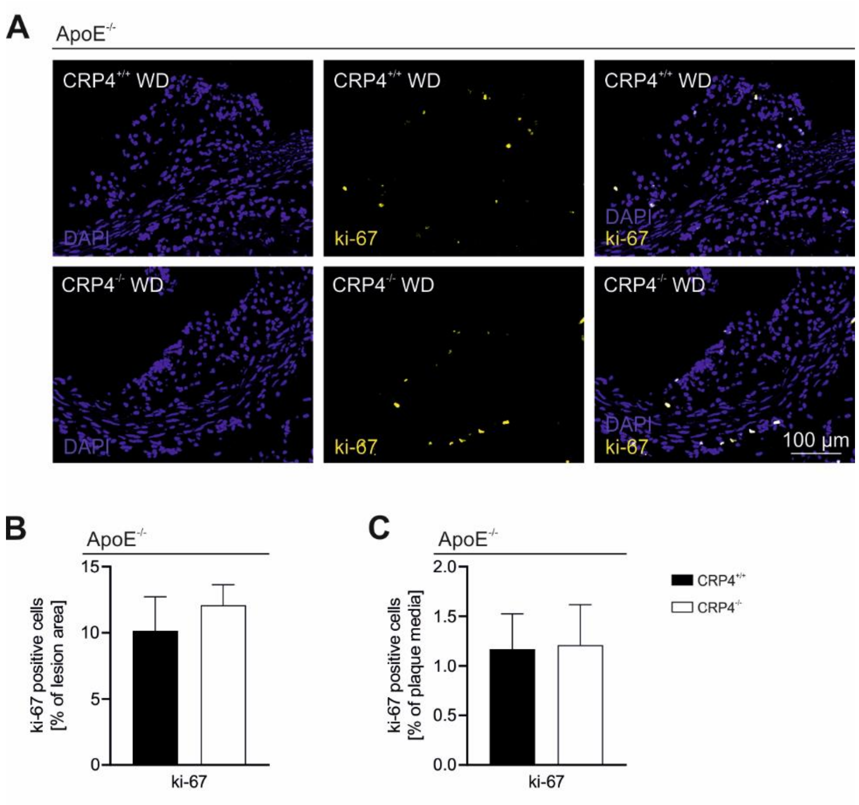

Supplement Figure S5. *Proliferation rate in ApoE-/- atherosclerotic plaques after 8 weeks of WD.*

A,B, The proliferation rate in atherosclerotic plaques was measured via IF-staining of the proliferation marker ki-67 (yellow), whereas nuclei were counterstained with Hoechst (blue). Ki-67-positive nuclei (%9 were slightly, but not significantly, higher in the lesion area of mice lacking CRP4. **C,** In the media the percentage of ki-67-positive nuclei did not differ between the genotypes, n=6 per genotype, two-tailed student t-test. All data expressed as means ± SEM.

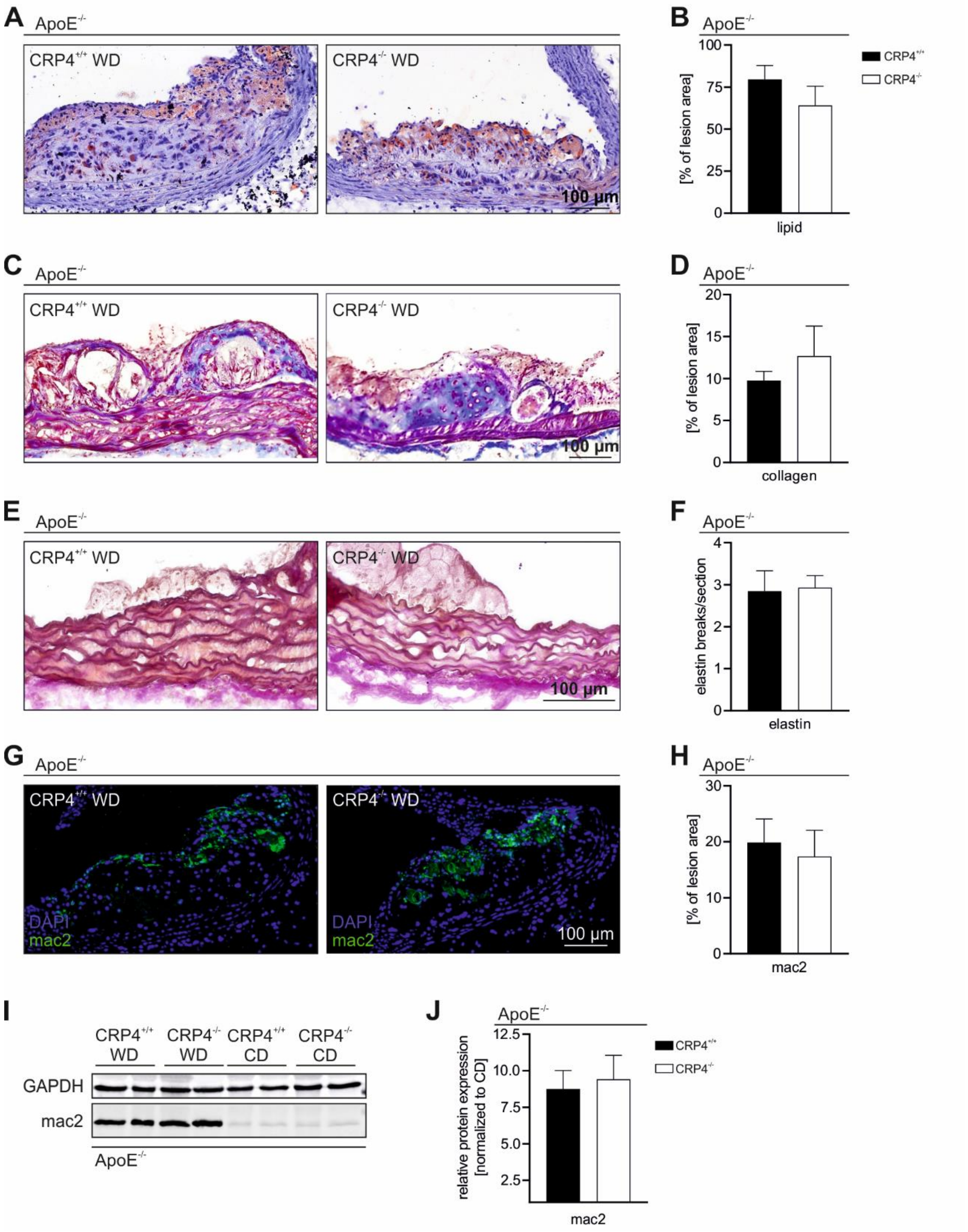

Supplement Figure S6. *Multiple plaque vulnerability parameters are not affected by CRP4.*

A, B, Oil red O staining and quantification of haematoxylin counterstained section from ApoE$^{-/-}$/CRP4$^{+/+}$ *versus* ApoE$^{-/-}$/CRP4$^{-/-}$ mice. At a histological level the amount of neutral lipids within the lesion area did not differ between genotypes. **C, D,** Collagen fibres were visualized by Masson trichrome staining and quantified. The amount of collagen in the lesion area was slightly, but not significantly, increased in CRP4-deficient mice. **E, F,** Plaque destabilizing elastin breaks were visualised via Elastika van Gieson staining. Breaks per section were not altered by the CRP4 status of the vessels. **G, H,** Macrophage-like cells were detected by using the mac2 antibody. Mac2^{+} cells accumulated in the lesion area of ApoE$^{-/-}$/CRP4$^{+/+}$ and ApoE$^{-/-}$/CRP4$^{-/-}$ vessels with no differences between genotypes, n=6-7

per genotype, two-tailed student t-test. **I, J,** Accordingly, the total amount of mac2+ macrophages in aortic lysates were significantly higher in WD compared to CD fed mice, but this increase was again not affected by the CRP4 status of the vessels, n=4 per genotype (every n was a pool of 2 aorta), two-tailed student t-test. All data were expressed as means ± SEM.

Supplement References

27. Straubinger, J.; Boldt, K.; Kuret, A.; Deng, L.; Krattenmacher, D.; Bork, N.; Desch, M.; Feil, R.; Feil, S.; Nemer, M., et al. Amplified pathogenic actions of angiotensin II in cysteine-rich LIM-only protein 4–negative mouse hearts. *The FASEB Journal* **2017**, *31*, 1620-1638, doi:https://doi.org/10.1096/fj.201601186.
